# Distributed Efficient Similarity Search Mechanism in Wireless Sensor Networks

**DOI:** 10.3390/s150305474

**Published:** 2015-03-05

**Authors:** Khandakar Ahmed, Mark A. Gregory

**Affiliations:** School of Electrical and Computer Engineering, RMIT University, Melbourne, VIC 3000, Australia; E-Mail: mark.gregory@rmit.edu.au

**Keywords:** Wireless Sensor Networks, distributed data centric storage, similarity search, range query, K-nearest neighbor query, sector based distance routing

## Abstract

The Wireless Sensor Network similarity search problem has received considerable research attention due to sensor hardware imprecision and environmental parameter variations. Most of the state-of-the-art distributed data centric storage (DCS) schemes lack optimization for similarity queries of events. In this paper, a DCS scheme with metric based similarity searching (DCSMSS) is proposed. DCSMSS takes motivation from vector distance index, called *iDistance*, in order to transform the issue of similarity searching into the problem of an interval search in one dimension. In addition, a sector based distance routing algorithm is used to efficiently route messages. Extensive simulation results reveal that DCSMSS is highly efficient and significantly outperforms previous approaches in processing similarity search queries.

## 1. Introduction

This paper considers a distributed information delivery and search service for one or more applications in a Wireless Sensor Network (WSN) that utilizes in-network storage, which is known as Data Centric Storage (DCS) [1]. The applications consist of a set of producer and consumer nodes that can exchange information by relaying packets through neighboring sectors. Nodes have no explicit knowledge of each other but are aware of the applications. The distributed information delivery and search service is used to implement an information delivery and search layer between applications and nodes that provides enhanced reliability and improved flexibility. This paper introduces Data Centric Storage with Metric based Similarity Searching (DCSMSS), which is a highly scalable distributed information service based on Disk Based Data Centric Storage (DBDCS) [2] that incorporates similarity searching. A data query search for an exact match or for data within a specified similarity range is called similarity searching. Similarity searching is particularly useful where users seek data within a WSN that is either a match or close to a match.

The member nodes in a sector or zone report the sensed event to their associated Sector Head (SH), which aggregates the received events at the end of each epoch (length of a Time Division Multiple Access (TDMA) slot assigned to each sector). The aggregated event is hashed to produce a hash key, which is mapped from a one dimensional domain into a metric space utilizing a normalized and adapted variant of *iDistance* [3]. The distance between a data point and its closest reference point plus a scaling value is called the point’s *iDistance*. In this paper distances between data points and reference points in the multi-dimensional space have been mapped to one-dimensional values.

The DCSMSS scheme presented is used to balance information transfer loads across the network, enhance reliability and provide efficient similarity searching within a distributed network for two types of queries—range query and *k*-query. DCSMSS uses a lightweight Sector Based Distance (SBD) routing algorithm, presented in [2,4], to route inter-sector storage, intra-sector storage and query traffic. The domain of the derived hash key of an aggregated sensed event, denoted by *H_D_*, is mapped into the metric space of the DBDCS architecture. In order to balance the load among the sectors, a pivot point generation procedure is used dividing *H_D_* into almost equally populated sub-intervals, denoted by *h_Di_*, where *h_Di_* ≠ *h_Dj_* and 0 ≤ *i* ≤ *j* ≤ S; *S* refers to the total number of sectors. In order to store an event, the target sector is mapped based on the derived hash key and pivot points. Furthermore, the target SH distributes the load among the member nodes based on the hash key value and distance to the member nodes.

The remainder of this paper is structured as follows: Section 2 provides an overview of the related work in the literature. Network architecture, data processing and mapping, SBD routing, insertion and querying are illustrated in Section 3. Section 4 describes the SBD analytical model. This is followed by the simulation results and performance evaluation of DCSMSS and SBD presented in Section 5. The paper is concluded in Section 6.

## 2. Related Work

A detailed literature survey that discusses key research on DCS techniques is presented in [1,5]. This section mentions researches, which are closely related to the research reported on in this paper. 

In order to process similarity search queries efficiently, Chung, *et al.* [6] propose a novel framework over a data-centric storage structure, referred to as the Similarity Search Algorithm (SSA), based on the concept of a Hilbert Curve. The lack of global knowledge about the entire sensor database is identified as one of the major challenges in processing a sensor network similarity search query. However, in order to overcome this constraint, SSA presents a network layout based on a Hilbert Curve, and hence, successfully avoids the need to collect data from all sensors when searching for the most similar data item. SSA divides the whole network recursively into *4^l^* square quadrants where *l* denotes the number of levels. The center (referred to indexing node and denoted by *I*) of each square quadrant (cell) is responsible for storing a sub-range of the entire range of an event denoted by *R* where *R_L_* and *R_U_* denotes the lower bound and upper bound, respectively. The data sub-range for which *I_ID_* (IDth indexing node) is responsible is denoted by (*R_L_*^ID^, *R_U_^ID^*) = (*R_L_* + (*I_ID_* − 1) × *r*, *R_L_* + *I_ID_* × *r*), where *n* × *r =* *R*, *n* is the number of indexing nodes. However, SSA is not applicable in multi-dimensional range queries or similarity searching. Furthermore, statistically 80%~90% of the reported sensed data points tend to be very close to the mean range. But, SSA divides the domain of an attribute into equal sub-ranges and maps each sub-range to a square quadrant. This design leads to an unbalanced load distribution across the network leaving most of the quadrants empty or lightly loaded compared to few heavily loaded quadrants. Finally, storing data only in the index node is highly inefficient due to its single point of failure and quicker depletion of storage space and energy than other nodes inside the cell.

Shen, *et al.* [7] propose an efficient spatial-temporal Similarity Data Storage (SDS) scheme for both static and dynamic WSN. In SDS, a deployed large scale WSN field is considered as a rectangular field. The entire field is divided into smaller rectangular zones (number of zones—horizontally *n_x_* and vertically *n_y_*). A node in the zone with *ID_i_* can calculate its Euclidean distance from another zone *ID_j_* using, with δ*x_i_*_,*j*_ = (*ID_i_* − *ID_j_*)%*n_x_* and δ*y_i_*_,*j*_ = (*ID_j_* − *ID_i_*)/*n_x_*. A WSN application data item is characterized by attributes referred to by *d*, and therefore, SDS uses Locality Sensitive Hashing (LSH) [8] to transform *d* to a series of hash values. For example, if a data item is characterized by *d*_1_ > *d*_2_ > *d*_3_, then their hash values conform to *h_d_*_1_ > *h_d_*_2_ > *h_d_*_3_, where *h_d_* is the hash value of *d*. In the mapping between data and zones, the *ID* differences between zones indicate the similarity between the data stored in the zones. 

Apart from the research mentioned above, a few other research contributions [9,10,11] provide current state-of-the-art implementations of range query mechanisms. 

In Multidimensional Attribute (MDA) [9], a data storage and range query method for multi-dimensional attributes in WSN is proposed. MDA is built on top of three assumptions: (1) The sensors are uniformly and densely deployed; (2) each node can sense multiple events; and (3) each node maintains a neighbor table *via* periodic beacon message exchanges and knows its own geographic location. After a source node detects an event *E* of *k* dimension, a *k*-bit referred to as a “*B*” code is assigned to it. *E* is then mapped to a range space *R* according to code *B*. In the data retrieval phase, a range query is split into a *k*-dimension range of multiple sub-queries. After splitting, each attribute sub-query is assigned with a bit code and tuples of *k*-bit codes, referred to as code “*B*”, are produced. The codes “*B*” are then mapped to range space and data is retrieved from nodes located in the mapped *R*.

Distributed Index for Features (DIFS) [10], performs a data fusion based on data conveyance through the network. The routing in DIFS is designed on top of a quad tree in a manner that balances the communication load across the index, and the range is maintained along the sensor node hierarchy. Unlike traditional binary and quaternary trees, DIFS constructs a multiply rooted hierarchical index, where a non-root node might have multiple parents. Information for a specific range within a particular geographic region is stored in corresponding nodes of that region. A node covering a large area stores a smaller range of values while a node covering a small area stores a wider range of values.

Li, *et al.* [11] proposed a method called Distributed Index for Multi-dimensional Data (DIM), which includes both a point and a range query in a multidimensional DCS model. In DIM, each sensor is linked as a node in a tree structure where each node represents a range of values. A root node represents the entire range of values and splits into two equal parts for left and right child nodes. This process continues for each non-leaf node until leaf nodes are reached. Table 1 summarizes related work with corresponding features.

**Table 1:** Summary of relevant DCS schemes.

	Mechanisms				Schemes
Range Query	Bit Code Mapping				MDA [9]
	Tree Structure	Multiply Rooted Hierarchical Index			DIFS [10]
		Binary Tree			DIM [12]
	Dimension (attribute)	Range Query *vs.* Point Query	Load Balancing	Data Aggregation	
Similarity Searching	Single	Both	Yes	No	SSA [6]
	Multi	Range	Yes	No	SDS [7]

## 3. Basic System Operation

### 3.1. Network Architecture

**Figure 1:**
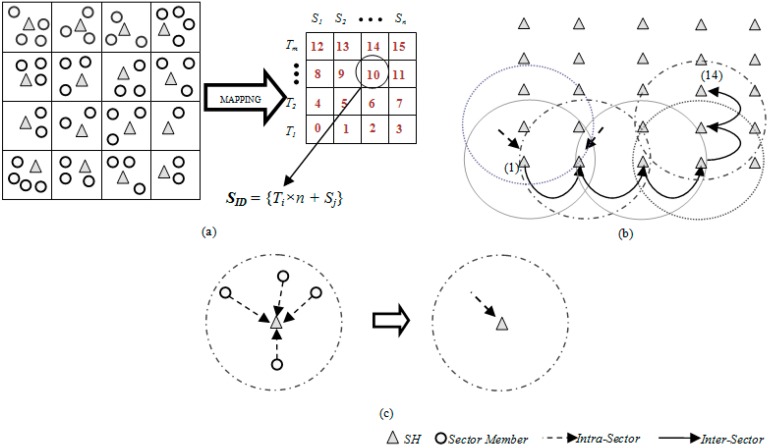
(**a**) DBS mapping; (**b**) inter-sector communication; and (**c**) intra-sector member node to head node communication.

The surface/platter of a magnetic disk storage device consisting of tracks and sectors provides an interesting approach that may be applied to a large scale WSN. This assumption led to a Disk Based Data Centric Storage (DBDCS) architecture, as shown in Figure 1a, dividing the rectangular field into a matrix of storage cells (referred to as a sector) where row and column represent track (*T_i_*) and sector (*S_j_*), respectively. In DBDCS, the covered network is considered as one of the storage surface and sector is considered as the core cell of storage. However, unlike magnetic storage disk, in DBDCS, the header file for data mapping is not located in one single particular location rather a dynamic mapping algorithm is used using hashing. Hence, each SH could calculate the target sector to read/write corresponding data. The physical deployment is mapped to an *m x n* matrix, where *m* is the number of tracks and *n* is the number sectors for each track. Hence, the nodes in the network are divided into *S* *(mxn)* sectors, each comprising a Sector Head (SH) and sector members that communicate *via* one hop to the SH (see Figure 1c), where *SH_i_* ϵ [1…*S*]. Each node is configured to be aware of the deployment layout by knowing: (1) All SHs are assigned with the sector number as a virtual address and node id, and (2) All member nodes know their own node id and number of tracks (*m*) and sectors (*n*) of the network field. As shown in Figure 1b, the intra-sector communication (*i.e.*, communication from sector members to *SH* or *vice-versa*) is constrained to one hop while inter-sector transmission is multi-hop. For simplification, the sensor nodes inside each sector are not shown explicitly in Figure 1b. Instead, an aggregated link (see Figure 1c) is shown to represent the total traffic from member nodes to head node. 

### 3.2. Metric-Based Searching

Metric space *M* can be defined as a pair *M* = (*D*, *d*), where *D* is the domain of objects and *d* is the *distance function—d:* *D* × *D* → **R** satisfying the following constraints for all objects *a*, *b*, *c* ϵ *D*:

*d*(*a*, *b*) ≥ 0 (non-negativity)
*d*(*a*, *b*) = 0 if *a =b* (identity)
*d*(*a*, *b*) = *d*(*b*, *a*) (symmetry)
*d*(*a*, *c*) ≤ *d*(*a*, *b*) + *d*(*b*, *c*) (triangle inequality) (1)

In this metric space, two types of similarity queries can be defined including range query *Range*(*q*, *r*) and *K-*nearest neighbor search *KNN*(*q*, *k*) by the resultant set *X*, considering I⊆D to be a finite set of indexed objects:

***Range*(*q*, *r*)**:
*X* = {*a* ϵ *I*|*d*(*q*, *a*) ≤ *r*} (2)

(3) $$KNN(q,k)\;X\subseteq I:\left|X\right|=k,\forall_{a}\in X,\forall_{b}\in I\backslash X:d(q,a)\leq d(q,b)$$

**Figure 2:**
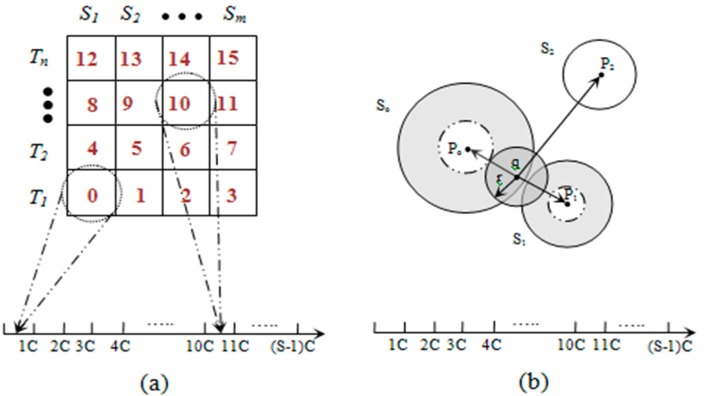
(**a**) Data mapping; and (**b**) range query example.

The data space can be divided into *S* segments (*S* is the total number of sectors) with a pivot point, denoted by *P_i_*, for each sector *S_i_*. The *iDistance* key for an object *x* ϵ *D* can be defined as (Figure 2a):

*iDist*(*x*) = *d*(*P_i_*, *x*) + *i*·*c* (4)

In Equation (4), *c* is the separating constant for individual sectors. Given *q* ϵ *D*, the range query for *q* with the range of *r* can be defined as (Figure 2b):

[*d*(*P*, *q*) + *i*·*c* − *r*, *d*(*P*, *q*) + *i*·*c* + *r*] (5)

In Equation (5), *q* denotes the query point and *P_i_* denotes the pivot point for *SH_i_* where *P_i_* ≤ *q* ≤ *P_i_*_+1_. Therefore, after locating the target sector (*SH_i_*), the conceptual range can be defined by Equation (5) and is illustrated in Figure 2b. The axis showed in Figure 2 represents the one dimensional data space that has been divided into *S* segments, where each sector is mapped to a segment.

### 3.3. Data Processing and Mapping

A sensed event *E* can be defined by an *l*-dimensional tuple, (*A*_1_, *A*_2_, *A*_3_, *…*, *A_l_*) where $A_{g},\forall_{g}\in \left[1,l\right]$ denotes the *g*th attribute and *D_Ag_* is the domain of attribute *A_g_*. Each member node of a sector transmits the sensed event as an *l*-dimensional tuple ${\langle v_{i1},v_{i2},......,v_{il}\rangle }_{k}$, where 1 ≤ *i* ≤ *M_k_*, *M_k_* is the total number of member nodes in the *k*th sector and *v_ij_* denotes the value of the *j*th attribute received from *i*th member node of the *k*th sector. The corresponding SH, after collecting tuples from all the member nodes, aggregates them at the end of each epoch before finding the target SH mapping*.* Hence, after aggregation at epoch *t*

(6) $$\begin{array}{ll} {\text{E}}_{\text{k}}\text{(Agg(t))} & =\;\int_{{\mathrm{Agg}}_{\text{i}=\text{1}}}^{M_{k}}\langle v_{i1},v_{i2},......,v_{il}\rangle \left(t\right)\\ & =\langle \psi_{1},\psi_{2},.......,\psi_{l}\rangle \left(t\right) \end{array}$$

(7) $$\begin{array}{l} \psi_{j}=\left\{\max_{i=1}^{M_{k}}v_{ij},\min_{i=1}^{M_{k}}v_{ij},avg_{i=1}^{M_{k}}v_{ij}\right\},\\ here,j\in \left[1,l\right],k\in \left[1,S\right] \end{array}$$

Here, it is assumed that the attribute’s aggregated values of ψ*_i_* have been normalized to be between the range of 0 and 1. From Figure 1a, lets consider 6th (*k* = 6) sector, where *M*_6_ = 3. If the total number of attribute is 3 then for any particular round (for example *t* = 2), Equation (6) can be illustrated as shown in Table 2.

**Table 2:** Illustration of Equations (6) and (7).

Member Node	First Attribute	Second Attribute	Third Attribute
1	*v*_11_	*v*_12_	*v*_13_
2	*v*_21_	*v*_22_	*v*_23_
3	*v*_31_	*v*_32_	*v*_33_
*After applying Equations (6) and (7)*			
	*max* (*v*_11_, *v*_21_, *v*_31_)	*max* (*v*_12_, *v*_22_, *v*_32_)	*max* (*v*_13_, *v*_23_, *v*_33_)
	*min* (*v_11_*, *v*_21_, *v*_31_)	*min* (*v*_12_, *v*_22_, *v*_32_)	*min* (*v*_13_, *v*_23_, *v*_33_)
	*avg* (*v*_11_, *v*_21_, *v*_31_)	*avg* (*v*_12_, *v*_22_, *v*_32_)	*avg* (*v*_13_, *v*_23_, *v*_33_)

As shown in Table 3, weights have been assigned to different attributes based on their importance in the event description. Hence, an attribute with higher weight has greater influence on the similarity among events.

**Table 3:** Weight settings.

Attribute	Weight
*A*_1_	*W*_1_
*A*_2_	*W*_2_
*....*	....
*A_l_*	*w_l_*

#### 3.3.1. Pivot Point Generation

**Figure 3:**
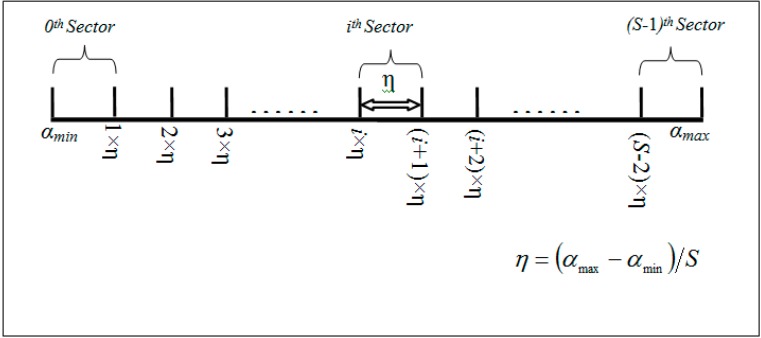
Pivot point generation example.

The domain of the one dimensional derived hash key *H_D_* of an aggregated *l*-dimensional sensed event can be defined by α (α_min_, α_max_) as illustrated in Figure 3. In Equations (8)–(11), A*_i_*_(min)_, A*_i_*_(max)_, A*_i_*_(avg)_ and A*_i_*_(θ)_ denote the minimum, maximum, average and threshold value of *i*th attribute. The center of mass (COM), denoted by β, is derived in Equation (10) to find the normalized center point of the domain of the hash key *H_D_* whereas δ is the separating factor between two pivot point. However, in order to balance the load among sectors, it is important to find the range where the concentration of the data points is high. Hence, β and δ can be used to find this COM range, denoted by β (β*_range_*_-min_, β*_range_*_-max_) as shown in Equation (12):

(8) αmin=∑i=1l((Αi(min)Αi(max))×wi)

(9) αmax=∑i=1l((Αi(max)Αi(max))×wi)

(10) β=∑i=1l((Αi(avg)Αi(max))×wi)

(11) δ=∑i=1l((Αi(θ)Αi(max))×wi)

(12) $$\begin{array}{l} \beta_{range-\min}=\beta -\delta \\ \beta_{range-\max}=\beta +\delta \end{array}$$

Thus, the separating step, denoted by η, between two pivot points in the COM range can be defined by:

(13) η=(βrange−max−βrange−min)S−1

Thus the pivot points for ***S*** sectors can be defined in each sector head by (Algorithm 1):

(14) $${Ρ}_{i}=\{\begin{array}{l} \alpha_{\min},\\ \beta_{range-\min}+i\times \eta ,\\ \alpha_{\max}, \end{array}\begin{array}{l} i=0\\ 0<i<S\\ i=S \end{array}$$

#### 3.3.2. Mapping

Given *l* attributes in an attribute list associated with weight *w_j_* (1 ≤ *j* ≤ *l*) in a WSN application, the source *SH_k_* generates the hash value by:

(15) $$h=\sum_{j=1}^{l}\left(\left(avg_{i=1}^{M_{k}}v_{ij}/A_{j(\max)}\right)\times w_{j}\right)$$

Hence, after each epoch, *SH_k_* forwards the aggregated event $E_{k}=\langle \left[\psi_{1},\psi_{2},\ldots ,\psi_{l}\right],\left[t,h\right]\rangle ,$ where *t* denotes the epoch number, to the destination sector head denoted by *SH_i_* where, *P_i_* ≤ *h* ≤ *P_i_*_+1_ and *P_i_* and *P_i_*_+1_ is the lower and upper limit of *i*th sub-interval, respectively.

### 3.4. SBD Routing

**Figure 4:**
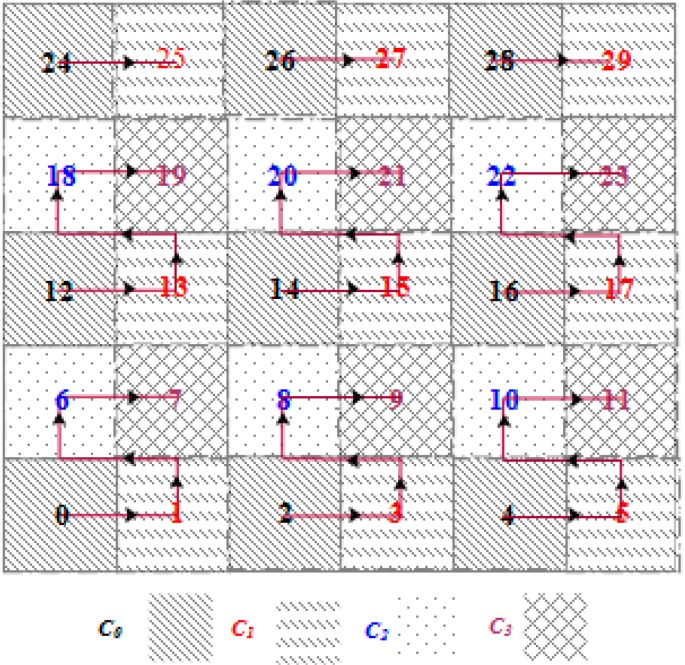
Slot assignment using algorithm 2 (GCA).

In order to relay aggregated packets from *SH_k_* to *SH_i_*, DCSMSS uses the Sector Based Distance (SBD) routing algorithm [4]. Each round of SBD consists of two phases: (a) Learning phase and (b) Relaying phase. The learning phase is again divided into three stages: (I) Sector head TDMA slot assignment stage using the grid coloring algorithm (GCA); (II) Member-SH association stage; and (III) Intra-sector TDMA slot assignment stage for member nodes managed by the SH*.* In the first stage of the learning phase, each SH finds the non-overlapping operating slot for corresponding sectors using Algorithm 2. It is assumed that each SH is configured to be aware of the number of sectors in the deployment layout. Using Algorithm 2, all sectors of any grid size could be assigned with conflict-free TDMA slot by reusing only four time slots. For example, Algorithm 2 has been applied to a grid of 30 sectors (see Figure 4). Each sector of the grid is assigned with conflict free time slot by reusing only four time slots (*C*_0_~*C*_3_). Sectors with similar time slot can perform concurrently without any interference. 

**Algorithm 1.** Pivot Point Generation Algorithm (implemented at each *SH* node).

**Input**: *attrRangeTable*  (containing minimum, maximum, average and theta of each attribute), *W* (weights to different attributes based on their importance in the event description).
**Output**: P (derived pivot point for each sector)
1: *mapRec.minRange* ← 0; *mapRec.maxRange* ← 0
2: *m* ← **lengthof**(*attrRangeTable*)
3: **for** each *i* from 1 to *m* **do**
4:	 *mapRec*.*minRange* ← *mapRec*.*minRange* + (*attrRangeTable*[*i*].*min*/*attrRangeTable*[*i*].*max*) × *W*[*i*]
5:	 *mapRec*.*maxRange* ← *mapRec*.*maxRange* + (*attrRangeTable*[*i*].*max*/*attrRangeTable*[*i*].*max*) × *W*[*i*]
6:	 *mapRec*.*com* ← *mapRec*.*com* + (*attrRangeTable*[*i*].*avg*)/*attrRangeTable*[*i*].*max*) × *W*[*i*]
7:	 *mapRec*.*theta* ← *mapRec*.*theta* *+* (*attrRangeTable.theta*)/*attrRangeTable*[*i*].*max*) × *W*[*i*]
8:	 *i* ← *i* + 1
9: **end for**
10: *comLowerLimit* ← *mapRec*.*com* − *mapRec*.*theta*
11: *comUpperLimit* ← *mapRec*.*com* + *mapRec*.*theta*
12: // *S* is the total number of sectors
13: η ← (*comUpperLimit* − *comLowerLimit*)/(*S* − 1)
14: **for** each *j* from 0 to *S* **do**
15: 	 **if** *j* = 0
16:	   **then** *P*[*j*] ← *mapRec*.*minRange*
17:	 **else if** *j* = *S*
18:	   **then** *P*[*j*] ← *mapRec*.*maxRange*
19:	 **else**
20:	   *P*[*j*] ← *comLowerLimit* + *j* × η
21:	 **end if**
22:	 *j* ← *j* + 1
23: **end for**



Hence, the frame length, denoted by *L*, of a round can be defined as:

*L* = 4 × ∆*t* (16.a)

∆*t* = |*C_i_*|, 0 ≤ *i* ≤ 3 (16.b)

Here, ∆*t* is the length of the TDMA time slot assigned to each sector.

**Algorithm 2.** Conflict free TDMA frame slot assignment GCA (implemented at each *SH* node).

**Input**: *H_D_* = 2 (circular hop distance between two sectors), *m*, *n* (total number of tracks (or rows) and sectors (or columns) in the grid, respectively)
**Output**: Conflict*-*free time*-*slot (*C_i_*) *with* frame length *L* = 4 × *epoch* (length of the slot assigned to a sector)
1:      **for** each *j* from 1 to *m* **do**
2:        **for** each *i* from (*j* − 1) × *n* to (*j* × *n* − 1) **do**
3:          **if** *i* < *n* × *j*
4:           **then** *SH_i_* ← *C*_0_
5:          **end if**
6:          **if** *i +*1 < *n × j*
7:           **then** *SH_i_*_+1_ ← *C*_1_
8:          **end if**
9:          **if** *i* + *n* < *m* × *n*
10:         **then** *SH_i+n_* ← *C*_2_
11:        **end if**
12:        **if** *i* + *n* + 1 < *m* × *n*
13:         **then** *SH_i+n_*_+1_ ← *C*_3_
14:        **end if**
15:        *i* = *i* + *H_D_*
16:      **end for**
17:      *j* = *j* + *H_D_*
16:    **end for**



In the Member-SH association stage, SH broadcasts a beacon frame and a member could receive beacon messages from more than one SH. Each member node then sorts the received beacon frames that come from more than one *SH* node based on Received Signal Strength Indicator (RSSI) into vector ν(*SH_i_*, *RSSI_i_*), where *RSSI_i_* ≥ *RSSI_i_*_+1_. In the presence of channel noise, fading and attenuation, it is not always possible to estimate the closest SH using RSSI only. Hence, in order to accurately find the closest *SH*, the round trip time (RTT) method has been used as well. According to this method, each Member Node (MN*)* sends a packet request to all candidate SHs in the list and waits for an immediate acknowledgment. After receiving the acknowledgment the MN calculates the distance of the corresponding SH from time of flight (TOF). It then calculates a ranking number for each candidate SH based on both RSSI and TOF and selects a SH from the candidate list that has highest ranking (see Algorithm 3).

According to this method, the time of flight, referred to as *T_TOF_* is calculated as follows

(17) $$T_{TOF}=\frac{T_{RTT}-T_{TCP}}{2}$$

Here, *T_RTT_* = Round Trip Time of Flight. *T_TCP_* = Time to Compute Packet.

The distance between two nodes can be calculated as

*d_RTT_* = *T_TOF_* × *c* (18)

Here, *c* = Speed of Light

The Equation (18) can further be rewritten after adding the faultiness as [13]:

(19) $$d_{RTT}=d+\varepsilon_{RTT}^{LOS}+\varepsilon_{RTT}^{NLOS}$$

Here, $\varepsilon_{RTT}^{LOS}$ = Error occurs for ranging in a line of sight setting. $\varepsilon_{RTT}^{NLOS}$ = Error due to ranging in a non-line of sight environment.

The negative impacts of multipath effects, a big factor, in $\varepsilon_{RTT}^{LOS}$ can be minimized using an empirical approach [14]. Uncertainties and noise in the hardware especially jitter effects play a key role in $\varepsilon_{RTT}^{NLOS}$. Considering the jitter component *T_TOF_* can be calculated as [15]

(20) $$T_{TOF}=\frac{T_{RTT}-T_{TCP}-(J_{t1}+J_{c1}+T_{TCP}+J_{c2}+J_{t2})}{2}$$

(21) $$T_{RTT}=J_{t0}+J_{c0}+TOF_{R}+TOF_{A}+J_{t3}+J_{c3}+T_{TCP}$$

In Equations (20) and (21), *TOF_R_* = TOF for the request packet. *TOF_A_* = TOF for the acknowledgment packet. *J_tN_* = jitter caused by the clock of transceiver. *J_cN_* = jitter caused by the clock of microcontroller.

The timestamps that are used to calculate the time between sending a request packet and receiving an acknowledge packet contain the jitter values *J_t_*_0_, *J_c_*_0_, *J_t_*_3_ and *J_c_*_3_. Another two timestamps that are considered in calculating the computation time between receiving a packet and sending the first bit of the ACK packet contain the jitter values *J_t_*_1_, *J_c_*_1_, *J_t_*_2_ and *J_c_*_2_.

The *MNs* then calculate the rank matrix for each candidate *SH* as

(22) ranki=(RSSIimaxi=1MN(RSSIi))+dRTTimaxi=1MN(dRTTi)

In Equation (22), *M_N_* is the total number of member nodes in *N*th sector.

The *MN*s, then send an association request to the *SH*, which has the highest rank in its list. This ensures the association of a member node to its closest head node (see Algorithm 3).

**Algorithm 3.** Head_Selection (), implemented in member nodes, selects the closest SH based on the rank calculated using Equation (22).

**Input:** *rank*, *SHInfo*
1:    **sort** *SHInfo* in descending order based on *rank*
2:    **create** network layer packet *joinCntrlPacket*
3:     *SH_D_* ← **pop** top element from *SHInfo.SH_S_*
4:     **set** *SELF_NET_ADDR* as source, *SH_D_* as destination and Packet Type = 4 to *joinCntrlPacket*
5:     //Unicast joining request to the closest head node.
6:     **toMacLayer** (joinCntrlPacket, SH_D_)



The SHs create a child table listing all the member nodes from which they receive association request. In the third stage of the learning phase, SHs broadcast a packet containing *C_k_* (0 ≤ *k* ≤3), ∆*t* and an array γ, where γ = {*m*_1_, *m*_2_, *m*_3_, …, *m_i_*} and |γ| = *M_k_*. In γ, *m_i_* and *i* denote the member node ID and index of this member node in the array, respectively. Each member node then calculates the intra-sector transmission slot based on their position in the array γ by:

(23) $$t_{i}=\{(i\times \ell )+(C_{k-1}\times \Delta t)|\gamma \left[i\right]==M_{S-ID},\gamma \left[i\right]\neq \gamma \left[j\right]\}$$

In Equation (23), ℓ and *M_S-ID_* are the length of the intra-sector TDMA time slot and the node’s self-network address, *i.e.*, node’s self-ID, respectively. The number of member nodes in a sector varies due to the dynamic nature of the Member-SH association procedure. Hence, the length of an intra-sector *TDMA* time slot can be defined by:

(24) ℓ=Δt(sizeof(γ)+1)

In the relaying phase, all member nodes report their buffered or aggregated sensed data to their associated SH during their allocated intra-sector TDMA transmission slot. A SH, after each epoch, *i.e.*, after collecting data from all member nodes, forwards the mapped event data (according to Section 3.2) in a multi-hop fashion to the corresponding sector for storage. In this inter-sector communication, SHs continue forwarding their packets to their immediate neighbor SH, which lies on the same row in the virtual grid (Figure 1a) until the packet reaches the SH that is on the same column as the destination sector. The packet is then forwarded vertically up or down until it reaches the destination (Figure 1b). The same process of routing is followed for query request and response. A description of the next hop selection process or algorithm during the relaying phase is given in Algorithm 4, which facilitates the selection of next hop in inter-sector communication. SHs continue forwarding their packets to their immediate neighbor in the same track until the packet reaches the same column where the destination sector lies. The packet is then routed vertically up or down until it reaches the destination.

A SH calls Algorithm 4 while acting as either: (I) a relaying node (receives a packet from MAC layer) or (II) a source node (receives packet from application layer).

**Algorithm 4.** Search_Next_Hop (*SH_i_*), implemented at each sector head node.

**Input**: Target *SH_i_*, where *SH_i_* ∈ [1...*S*], *m*- number of tracks (rows) and *n*- number of sectors per track (columns)
**Output**: Next Hop *SH_k_*, where *SH_k_* ∈ [1...*S*],
1:   //Finding the row and column position of //destination sector head and current head in the //grid
2:   destCol ← *SH_i_*%*n*;
3:   destRow ← *SH_i_*/*n*;
4:   curCol ← nextHopCol ← (SELF_NET_ADDR)%*n*
5:   curRow ← nextHopRow ← (SELF_NET_ADDR)/*n*
6:   *SH_k_* ← −1
7:    //Moving the packet to the same column where //destination sector lies
8:    **if** curCol < destcol
9:       /*Move toward right */
10:     **then** nextHopCol ← nextHopCol + 1 
11:   **else if** curCol > destcol
12:     /*Move toward left */
13:     **then** nextHopCol ← nextHopCol − 1
14:   //It is in same column so move toward up or down
15:   **else if** curCol = destCol
16:     **then** **if** curRow < destRow
17:       /*Move vertically up*/
18:       **then** *nextHopRow* ← *nextHopRow* + 1
19:     **else if** curRow > destRow
20:       /*Move vertically down*/
21:       **then** *nextHopRow* ← *nextHopRow* − 1
22:     **end if**
23:  **end if**
24:  /*convert to sector number*/
25:  *SH_k_* ← *nextHopRow* × *n* + *nextHopCol*
26:  **Return** *SH_k_*



#### Alternate Route

In the case of any primary route failure (*first travels toward a track and then a sector*), SBD switches to *recovery mode* of operation. In *recovery mode*, SHs follow alternate route:

Case 1: Route interruption along **track** path

(a) The last relay *SH_R_* forwards the packet one hop up or down along **sector** path.
(b) SBD returns to its normal mode of operation.

Case 2: *Route interruption along **Sector***

(a) The last relay *SH_R_* forwards the packet one hop left or right along the ***track*** path
  (I) The recipient *SH_R_* forwards the packet up or down along ***sector*** path
(b) SBD returns to its normal mode of operation.

**Figure 5:**
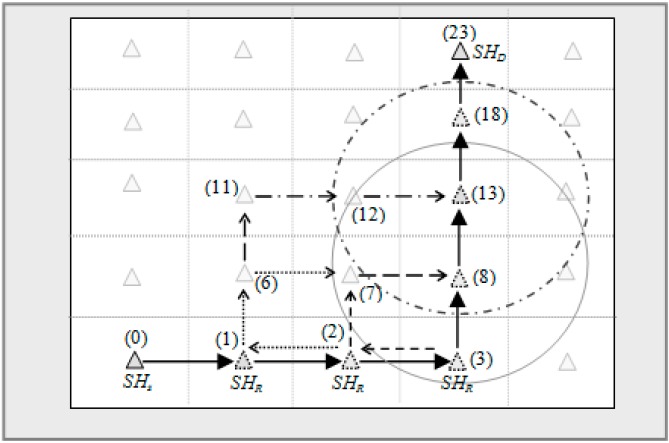
Some of the possible alternate routes from *SHs* → *SH_D_*.

For example, Figure 5, the source of the packet is *SH*_0_ and destination is *SH*_23_. Hence the primary and possible routes of transmission are:

(0) →(1) → (2) → (3) → (8) → (13) → (18) → (23)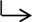 (7) → (8) → (13) → (18) → (23)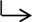 (1)→ (6) → (7) → (8)→(13) → (18) → (23)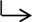 (11) → (12) → (13) → (18) → (23) and so on.

### 3.5. Insertion

**Figure 6:**
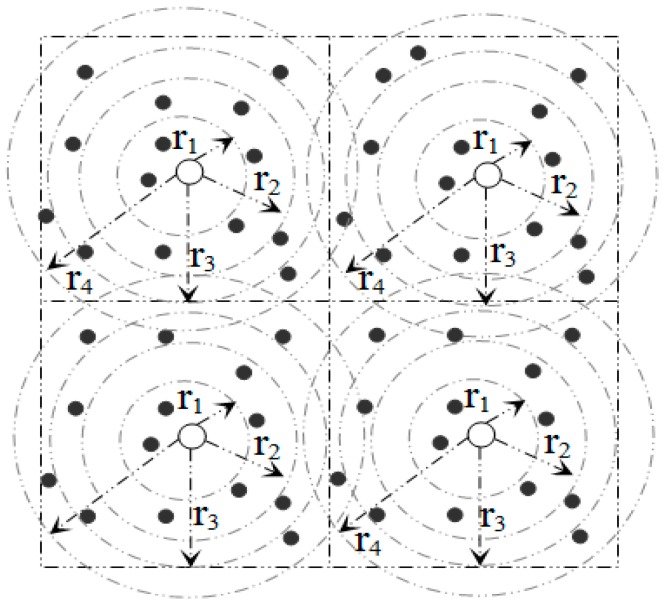
Formation of balls or segments inside a sector.

**Figure 7:**
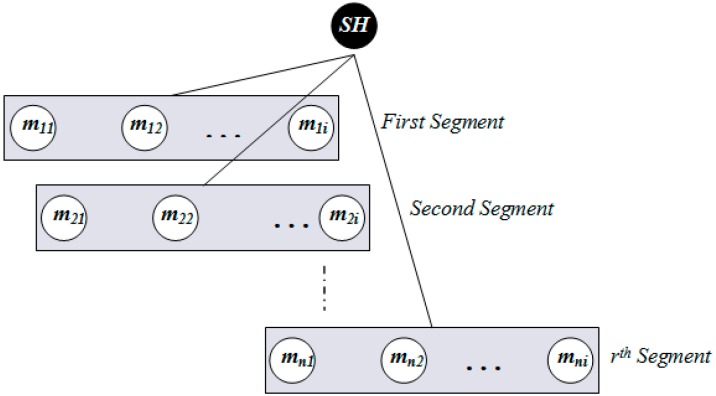
Segmentation architecture of member nodes inside a sector.

Within a sector, data is further distributed among nodes according to their distance from the SH. To do this, a sector is divided into segments. Figure 6, Figure 7 and Table 4 illustrate the idea of sector segmentation. Given a *k*th sector containing *M_k_* member nodes, the *SH_k_* first sorts all member nodes based on RSSI in ascending order. The member nodes are then divided into *r* segments. Each segment forms a ball, denoted by *B_(X_*_,*Y)*_ (*r_i_*), where the ball centered in (X, Y) of radius *r_i_*. (X, Y) is the geographic co-ordinates for *SH_k_*. The number of segments depends on the WSN application, the size of a sector and the number of member nodes in each sector. Thus the set of sensors that are within a Euclidean distance *r_i_* from (X, Y) form the segment defined by:

(25) $$B_{\left(X,Y\right)}(r_{i})=\left\{SensorsCoordinate\left(x,y\right):\left|\left(X,Y\right),\left(x,y\right)\right|\leq r_{i}\right\}$$

(26) $$\beta_{k}=\left({Ρ}_{k+1}-{Ρ}_{k}\right)/r,\begin{array}{cc} & 1\leq k\leq S \end{array}$$

(27) $${\left\{{Ρ}_{M_{K}(i)}\right\}}_{k=1}^{S}=\{\begin{array}{l} {Ρ}_{k},\\ {Ρ}_{k}+\beta \times i,\\ {Ρ}_{k+1}, \end{array}\begin{array}{l} i=0\\ 0<i<r\\ i=r \end{array}$$

**Table 4:** Member table of a SH node.

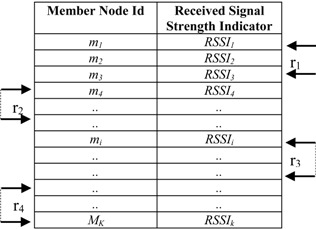

By Equations (26) and (27), the pivot points for *r* segments within the *k*th sector are calculated. An event with hash value, denoted by *h*, is stored in a member sensor node of *i*th segment where ${Ρ}_{M_{K}(i)}\leq h\leq {Ρ}_{M_{K}(i+1)}$. In order to balance the load, data is distributed among the nodes inside a segment in a round robin fashion (see Algorithm 5).

**Algorithm 5.** Search_Target_Node (*segment*[*i*]), implemented at each *SH* node.

  **Input**: *segment*[*i*] (a data structure containing member node ID and tally to count the number of packets stored in the corresponding member node)
  **Output**: return the target Member Node ID.
  1: **sort** *segment*[*i*] in ascending order based on *segment*[*i*]*.tally*
  2: *segment*[*i*].tally ← *segment*[*i*].*tally* + 1
  3: *memberNodeId* ← segment[*i*].*ID*
  4: **return** *memberNodeId*



### 3.6. Querying

#### 3.6.1. Range Query

Range query, issued by the query nodes from a sector of the network, denoted by *Range* (*q*, *r*), where *q* can be defined by an *l*-dimensional tuple, (*q*_1_, *q*_2_, *q*_3_, …, *q_l_*) where $q_{g},\forall_{g}\in \left[1,l\right]$, denotes the query value of the *g*th attribute and *r* is the weighted range to be considered. Hence, the query node first calculates

(28) $$h_{q}=\sum_{i=1}^{l}\left(\left(q_{i}/A_{i(\max)}\right)\times w_{i}\right)$$

where, *h_q_* denotes an aggregated query hash. The query range can be defined by [*h_q_* − *r*, *h_q_* + *r*]. The target head nodes where the query is to be forwarded are *SH_j_*, *SH_j_*_+1_, …, *SH_k_* where *P_j_* ≤ *h_q-r_* ≤ *P_j_*_+1_, *P_k_* ≤ *h_q+r_* ≤ *P_k_*_+1_ and *j* ≤ *k*.

*SH_j_* pulls data from the member nodes belonging to the segments: $B_{\left(X,Y\right)}\left(r_{t}\right),B_{\left(X,Y\right)}\left(r_{t+1}\right),...,B_{\left(X,Y\right)}\left(r_{r}\right)$ of the *j*th sector, where ${Ρ}_{M_{j}(t)}\leq h_{q-r}\leq {Ρ}_{M_{j}(t+1)}$ In contrast, *SH_k_* pulls packets from the member nodes belonging to the segments: $B_{\left(X,Y\right)}\left(r_{0}\right),B_{\left(X,Y\right)}\left(r_{1}\right),...,B_{\left(X,Y\right)}\left(r_{t}\right)$ where ${Ρ}_{M_{j}(t)}\leq h_{q+r}\leq {Ρ}_{M_{j}(t+1)}$. The rest of the head nodes: *SH_j_*_+1_, *SH_j_*_+2_, …, *SH_k−_*_1_ pull data from all of their member nodes.

**Figure 8:**
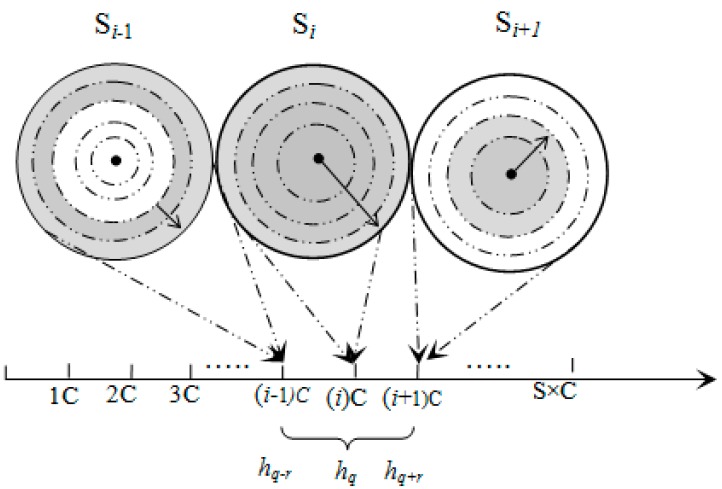
Range query example.

Suppose, Figure 8, *hq* is the hash value of the query (*q*, *r*). Hence, the range of the hash is [*h_q_ − r*, *h_q_ + r*], where *h_q_* − *r* belongs to (*i* − 1)*th* sector and *h_q_* + r belongs to *i* + 1th sector. Thus the target head nodes are (*i* − 1)th, *i*th, (*i* + 1)th sectors. Furthermore, within (*i* − 1)*th* sector, data is fetched from the member nodes of ${\left\{r_{i}^{th}\right\}}_{i=t}^{r}$ segments, where ${Ρ}_{M_{i-1}(t)}\leq h_{q-r}\leq {Ρ}_{M_{i-1}(t+1)}$. On the other hand, within (*i* + 1)*th* sector data is fetched from the sensor nodes of ${\left\{r_{i}^{th}\right\}}_{i=1}^{t}$ segments, where ${Ρ}_{M_{i+1}(t)}\leq h_{q+r}\leq {Ρ}_{M_{i+1}(t+1)}$. Finally, within *i*th segment, data is fetched from the whole sector.

#### 3.6.2. K-Nearest Neighbor Query

Like *range* query, a query node first calculates hash *h_q_* using Equation (28) for *K-nearest Neighbor Query* denoted by *KNN* (*q*, *k*). Here, *q* is defined by an *l*-dimensional tuple (*q*_1_, *q*_2_, *q*_3_, …, *q_l_*) where $q_{g},\forall_{g}\in \left[1,l\right]$, denotes the query value of *g*th attribute and *k* is the number of nearest neighbor nodes containing similar data to *q*. Thus the *KNN* (*q*, *k*) is first forwarded to the target sector head node, denoted by *SH_i_*, where *P_i_* ≤ *h_q_* ≤ *P_i_*_+1_. 

The *KNN* retrieval protocol is iterative. The SH scans through its segmentation table and includes the closest segment one after another until the following condition is true:

(29) $$\sum_{j=1}^{z}\Phi_{j}\geq k,\begin{array}{cc} where & z\leq r \end{array}$$

**Figure 9:**
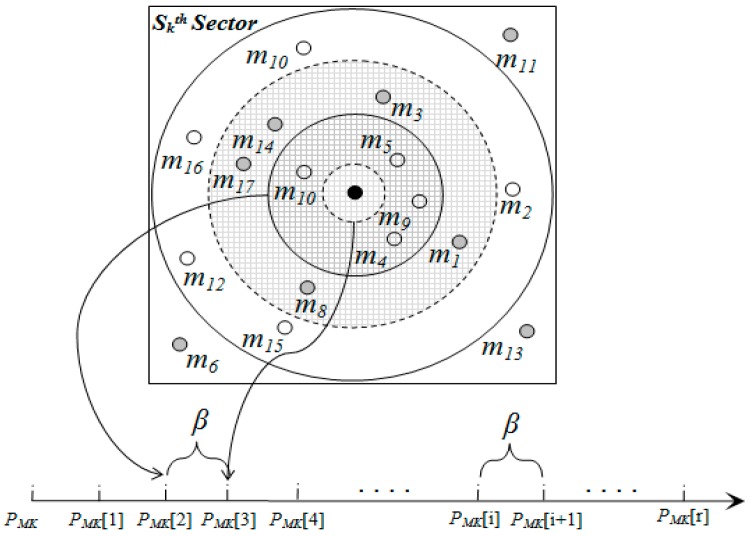
The *KNN*(*q*, 9)—nearest neighbor nodes containing similar data to *q* inside Sth sector are *m*_1_, *m*_3_, *m*_14_, *m*_17_, *m*_8_, *m*_10_, *m*_4_, *m*_9_, *m*_5_. Range Query Example.

In Equation (29), Φ_*j*_ denotes the total number of member nodes in the *j*th segment of the target *i*th sector. The *SH_i_* then broadcast a query request to all the member nodes of the ${\left\{j^{th}\right\}}_{j=1}^{z}$ segments. The member nodes of the corresponding segments respond to the query request. The *SH_i_* accumulates the received responses and sends them to the source query node. Figure 9 shows an example of *K-*Nearest Neighbor Query with the value of *K =* 9*.*

## 4. DCSMSS Analysis

### 4.1. SBD Analysis

This section analyzes the SBD performance in terms of routing message complexity (total number of message transfers in the network). The notations used in this section are summarized in Table 5. For simplicity it is assumed that the data transmission is error-free. Assume that the local sensor sampling and reporting rate to *SH* is *α*, the remote update rate is λ and the query rate is η. Let *C_lu_*, *C_ru_* and *C_qr_* be the cost of local update, cost of remote update and cost of getting an answer to a query, respectively. Hence, based on this assumption the overall message routing complexity can be defined as shown in Equations (30) and (31).

(30.a) $$C=\alpha \cdot C_{lu}+\lambda \cdot C_{ru}+\eta \cdot C_{qr}$$

(30.b) $$C=\alpha \cdot C_{lu}+\lambda \cdot C_{ru}+\eta \cdot 2\cdot C_{ru}$$

(31) $$C_{lu}=C_{tx}(r)$$

Here, *C_tx_* is the transmission cost by a wireless sensor node that covers a transmission range of rinside a sector.

For a single remote update issued by *S*_0_ (represented by track (*t*_0_) and sector (*s*_0_)) to *SH* in *S*_1_ (*t*_1_, *s*_1_), *S*_2_ (*t*_2_, *s*_2_), ..., *S_n_* (*t_n_*, *s_n_*), SBD sends out *n* updates to *n* different *SHs.* Let *C_ru,to,so,n_* and *C_tx,SH_* be the cost of remote update from *S*_0_ to *S*_1_, *S*_2_, ..., *S_n_* and transmission cost between two *SH*s, respectively. Hence, the cost of this remote update routing can be given by Equation (26).

(32) $$C_{ru,t_{0},s_{0},n}\leq \sum_{i=0}^{n}(\left|t_{0}-t_{i}\right|+\left|s_{0}-s_{i}\right|)\times C_{tx,SH}$$

**Table 5:** Notations used in analysis.

Notations	Description
C_lu_	Cost of local update
α	Local update rate
C_ru_	Cost of remote update
λ	Remote update rate
C_qr_	Cost of query request and response
η	Query rate
S_i_	*i*th sector number
t_i_	*i*th track number (row)
s_i_	*i*th sector number (column)
C_ru,to,so,n_	Cost of remote update from *S*_0_ to *S*_1_, *S*_2_, …, *S_n_*
*C_tx,SH_*	Transmission cost between two *SH*

For simplicity, consider that the data first travels toward a corresponding track and then sector. So, the longest distance the data travels up or down is:

(33) $$\max\left\{t_{0}-t_{1},.......,t_{0}-t_{n}\right\}\;\text{or}\;\left|\min\left\{t_{0}-t_{1},........,t_{0}-t_{n}\right\}\right|$$

Similarly the longest distance the data moves left or right is:

(34) $$\max\left\{s_{0}-s_{1},.......,s_{0}-s_{n}\right\}\;\text{or}\;\left|\min\left\{s_{0}-s_{1},........,s_{0}-s_{n}\right\}\right|$$

Before forwarding an update, it is possible to merge the packets having the same destination as their next hop and hence it is possible to optimize traffic. Thus, the horizontal and vertical routing cost can be minimized to:

(35) $$\begin{array}{l} \max\left\{t_{0}-t_{1},.......,t_{0}-t_{n}\right\}\\ +\left|\min\left\{t_{0}-t_{1},.......,t_{0}-t_{n}\right\}\right| \end{array}\;\text{and}\;\begin{array}{l} \max\left\{s_{0}-s_{1},.......,s_{0}-s_{n}\right\}\\ +\left|\min\left\{s_{0}-s_{1},.......,s_{0}-s_{n}\right\}\right| \end{array}$$

In the ideal situation the lower bound for the routing cost that can be achieved is:

(36) $$C_{ru,t_{0},s_{0},n}\geq \max\left\{t_{0}-t_{1},.......,t_{0}-t_{n}\right\}+\left|\min\left\{t_{0}-t_{1},.......,t_{0}-t_{n}\right\}\right|+\max\left\{s_{0}-s_{1},.......,s_{0}-s_{n}\right\}+\left|\min\left\{s_{0}-s_{1},.......,s_{0}-s_{n}\right\}\right|$$

Based on Equations (32) and (36) we find:

(37) $$\begin{array}{l} \max\left\{t_{0}-t_{1},.......,t_{0}-t_{n}\right\}+\left|\min\left\{t_{0}-t_{1},.......,t_{0}-t_{n}\right\}\right|+\max\left\{s_{0}-s_{1},.......,s_{0}-s_{n}\right\}+\left|\min\left\{s_{0}-s_{1},.......,s_{0}-s_{n}\right\}\right|\\ \leq C_{ru,t_{0},s_{0},n}\leq \sum_{i=0}^{n}(\left|t_{0}-t_{i}\right|+\left|s_{0}-s_{i}\right|)\times C_{tx,SH} \end{array}$$

Hence, *C_ru,to,so,n_* is at least equal to the lower or upper bound defined in Equation (37) and no lower than or greater than the respective bounds.

The producer *SH* node and target storage node are considered to be randomly distributed. It is assumed that all sectors have the same probability to disseminate updates. The remote update cost can be defined as:

(38) $$C_{ru}=\frac{\sum_{t_{i==0}}^{\sqrt{S}}\sum_{s_{i=0}}^{\sqrt{S}}C_{ru,t_{0},s_{0},n}}{S}$$

Here ***S*** is the total number of sectors.

## 5. Performance Evaluation

Simulations were conducted using Castalia v3.2 [16] running on top of OMNET++ [17] to evaluate the SBD and DCSMSS performance. The system parameters and their settings used in the experiments are summarized in Table 6. The network model (illustrated in Section 3.1) was tested in four rectangular fields with different parameter settings. Simulations were run 30~40 times with varying-channel affecting seeds to provide results that included average and 95% confidence interval. In Section 5.1, performance of SBD was tested in terms of Energy Consumptionand Latency. For the experiments presented in Section 5.1.1 and Section 5.1.2, the routing efficiency of SBD is evaluated against Low Energy Adaptive Clustering Hierarchy (LEACH) [18], Greedy Perimeter Stateless Routing (GPSR) [19], Directed Diffusion (DD) [20] and Car Pooling [7]. The querying performance of DCSMSS is evaluated in Section 5.2 in terms of Point Query, Range Query, KNN Query, Similarity Searching and Scalability. For the experiments presented in Section 5.2.1, Section 5.2.2, Section 5.2.3, Section 5.2.4 and Section 5.2.5, the querying performance of DCSMSS is evaluated against SDS, GHT [21] and DD.

**Table 6:** Simulation parameters.

Parameter	Setting
Field Size	60 × 60 m^2^, 90 × 90 m^2^, 120 × 120 m^2^, 150 × 150 m^2^
Number of Nodes (*n*)	80 (3600 m^2^), 180 (8100 m^2^), 320 (14,400 m^2^), 500 (22,500 m^2^)
Member Node Density (*f_m_)*	1 node/56.25 m^2^
Sector Head Node (SH) Density (*f_SH_)*	1 node/225 m^2^
Radio Range (member node)	~8 m
Radio Range (SH)	~20 m
Transmission Power	0 dBm (*SH*), –5 dBm (*membernode*)
Power Consumption in Sending and Receiving Messages	57.42 mW (*SH*), 46.2 mW (*member node*)
Power Consumption Per Sensing	0.02 mJoule
Data Rate, Modulation Type, Bits Per Symbol, Bandwidth, Noise Bandwidth, Noise Floor, Sensitivity	250 Kbps, PSK, 4, 20 MHz, 194 MHz, -100 dBm, -95 dBm
pathLossExponent	2.4
Initial Average Path Loss (*PL*(*d*_0_))	55
Reference Distance (*d*_0_)	1.0 m
Gaussian Zero-Mean Random Variable (*X*_α_)	4.0
MAC Protocol, Maximum Transimission Retries	SMAC [16], 2
SMAC Acknowledgment, Synchronization, RTS, CTS Packet Size	11, 11, 13, 13 bytes

Weight Matrix, and thus level of significance, is set using the configuration file that is used to initialize the network during the deployment of the network. In addition, an XML file is used that can be dynamically loaded any time from any SH and thus any change of the behavior of the environment or network can be disseminated throughout the network. The frequency of this dynamic dissemination technique is 1/round, where round = 1, 2, 3, ..., and this frequency is set based on how quickly the monitored network changes its behavior over time. The aggregation schemes are loaded at initialization of the network and can be changed on-demand during run-time. However, on-demand update during run time doesn’t effect on previously collected data.

It is obvious that LSH is a very powerful tool. However, LSH is good for data with high dimension. In WSN, dimension is usually limited and fixed at the time of deployment because total number of dimension depends on the number of sensor attached to a node. Thus a similarity searching based on the events, which are categorized in terms of attributes, is not scalable. In this paper, multi-dimensional data has been normalized into a one-dimensional domain. The domain is segmented into n intervals, where n is the total number of sectors. Each sector is responsible for storing data that falls in that interval. Hence, we could say, this hash function is more suitable than LSH for WSN.

### 5.1. SBD Performance

The performance of SBD is evaluated in comparison with DD, GPSR, LEACH and Car Pooling routing. The candidate routing protocols for evaluation were chosen from the literature based upon their being an acceptable representation of existing comparative techniques. DD, GPSR, and Car Pooling were used in different DCS schemes over the last decade. On the other hand, SBD, LEACH and Car Pooling are cluster routing algorithms. DD, a data-centric routing technique, floods the query to a region of interest that contains the data sought for. One of the widely used point-to-point routing algorithms is GPSR, which is used in earlier DCS schemes. GPSR implements two distinct routing algorithms—greedy forwarding algorithm and perimeter forwarding algorithm. Greedy forwarding algorithm moves packets progressively closer to the destination at each hop. At a void situation, where there is no greedy path, it switches to perimeter forwarding mode, in which a packet traverses consecutively closer along a planer sub-graph of the full radio network connectivity graph. This continues until it reaches to a node closer to the destination where greedy forwarding resumes. In LEACH and Car Pooling, sensor nodes are grouped into clusters with a Cluster Head (CH) for each group. A CH is responsible for data aggregation and communicating with other CH on behalf of the cluster nodes. However, unlike LEACH, in Car Pooling routing, the next hop is determined from the neighbor head node, which is closest to the destination head node. Nevertheless, packets with a common next hop are aged and sent together in order to reduce overhead though they might have different destinations. The consequent sub-sections present the performance evaluation of SBD in terms of Energy Consumption, Reliability and Latency against Car Pooling, LEACH, GPSR and DD.

#### 5.1.1. Energy Consumption

**Figure 10:**
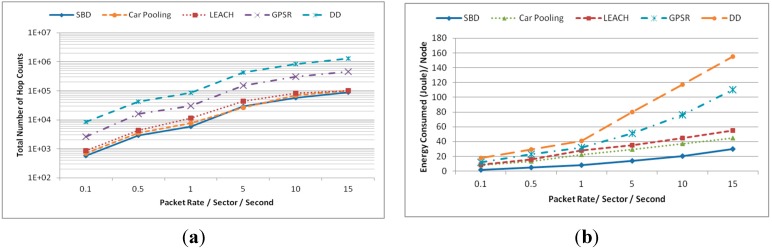
(**a**) Average energy consumption per node (joule) and (**b**) total number of hops (number of hops in storage and query routing).

**Figure 11:**
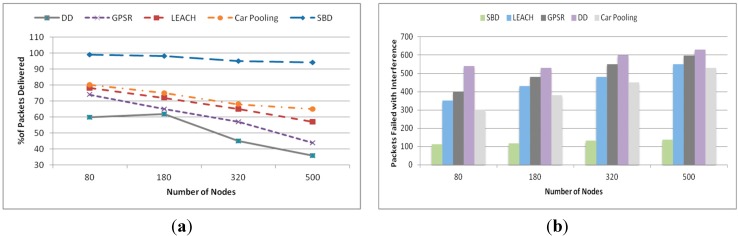
(**a**) Percentage of packets successfully delivered and (**b**) number of packets that failed due to interference.

This experiment was conducted in a network of 180 nodes in a 90 m × 90 m (8100 m^2^) field with a simulation time of 60 s. The data production and consumption rate per sector was varied between 0.1~15 packets per second. Figure 10a,b show the average energy consumption (joules) per node and total number of hop counts, respectively, as a function of packet rate per sector per sec. As shown in Figure 10a, SBD exhibits the lower energy consumption in all cases (low to high traffic rate). On the contrary, the energy consumption and total number of hop counts of DD are significantly higher than other methods and grows sharply due to its broadcasting. Figure 10b shows an interesting contrast. As shown in Figure 10b, the total number of hops for SBD, LEACH and Car Pooling is almost the same due to their similar clustering nature. However, despite having similar hop counts SBD outperforms all other approaches in energy consumption because SBD employs GCA to allocate conflict free scheduling. This helps to avoid packet retransmission as the chances of packet loss due to interference or collision is very low (see Section 5.1.2, Figure 11b).

#### 5.1.2. Latency

**Figure 12:**
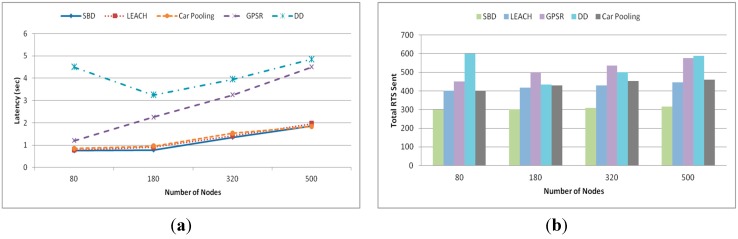
(**a**) Latency (sec) and (**b**) total RTS sent.

The setting for this experiment was the same as for the reliability experiment except for the total number of remote storage updates and queries, which were set to 100 each (generating 300 application packets including 100 storage updates, 100 query requests and 100 query responses). Figure 12a shows the latency of each method. Here, latency is defined as the time from the source sending a remote packet (storage update/query/response) to the destination receiving it. As expected, the latency of each method increases gradually with the increase in network size except for one case. It is observed that DD leads to the highest latency with a higher value than the other methods especially when there are 80 nodes. This happened because DD broadcasts 100 queries among the small number of nodes, which makes it more likely to generate congestion. LEACH, SBD and Car Pooling show similar low latency. Figure 12b depicts an interesting explanation for the result provided in Figure 12a. In Figure 12b, it is noted that the number of total Request to Send (RTS) sent by SBD is almost equal to the number of remote packets (remote update, query and response) while for DD it is almost a factor of two and for the other algorithms it is one and a half. However, despite having lower packet loss and lower retransmission compared to LEACH and Car Pooling, SBD shows similar latency due to its store and forward technique.

### 5.2. Querying Performance

In this section, the performance of DCSMSS with SBD and LEACH routing algorithm is evaluated in comparison with SDS, DD and GHT. As mentioned earlier, DD broadcasts a query to search for all of the desired data. GHT applies a hash function on the attribute name to find the location of the data and merges the located data to the query result. SDS uses the Locality Sensitive Hash (LSH) function and the number of hash values for a data item after the LSH operation was set to 5.

#### 5.2.1. Point Query

This experiment was conducted to evaluate the performance of each approach for point queries, which returns a single data item if it finds an exact match. The experiment was conducted using a 90 m × 90 m rectangular field, in which 180 nodes were randomly and independently disseminated. 300 queries, in total, were generated uniformly from different parts of the network. Queries were generated as a group referred to as a *batch*, which is sent out at the same time. The next group was released once all the queries of the previous batch were resolved or the maximum response waiting time was exceeded.

**Figure 13:**
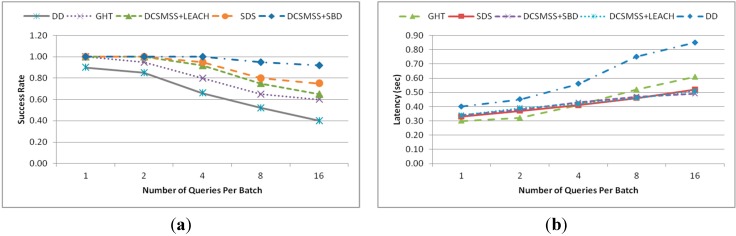
(**a**) Success Rate and (**b**) latency.

Figure 13a shows the success rate of different methods. Success rate is defined as the ratio between the number of successfully resolved queries and the total number of queries generated. This metric is used to reflect the effectiveness of a data storage method. From Figure 13a, it is observed that DD exhibits the worst performance and its success rate falls sharply as the number of queries per batch increases. With increased number of queries per batch DD’s broadcasting causes excessive messages, which leads to congestion and high packet loss. DCSMSS+SBD maintain a low packet loss due to its collision avoidance technique. The other three approaches—GHT, DCSMSS + LEACH and SDS fall in the middle. However, amongst these three, GHT’s performance is slightly lower. GHT routing uses a node as a step unit rather than zone or sector. As a result, it leads to a bit higher traffic causing more congestion and packet loss than those of the DCSMSS + LEACH and SDS.

Figure 13b shows that DD’s latency grows radically due to the congestion as traffic increases. Since DD uses broadcasting for data querying, it produces excessive message and traffic congestion when the number of queries per batch increases. DCSMSS + SBD, DCSMSS + LEACH and SDS have almost similar latency. GHT takes the shortest path and thus it outperforms other approaches when the traffic was low but its latency is affected by the congestion caused by the increased traffic with the increase in the number of queries per batch. DCSMSS and SDS schemes do not need to send as many queries as GHT since they rely on neighbor zone or SH to forward queries, thus reducing traffic and congestion. DCSMSS + SBD produces less traffic by realizing the collision avoidance technique (GCA) compare to DCSMSS + LEACH and SDS with Car Pooling. Due to the collision free time slot allocated to SH in the routing layer through GCA, SBD in DCSMSS uses a store and forward technique. However, the overhead that was added due to the store and forward technique is consistent regardless of traffic volume. Hence, it is observed from Figure 13b that SBD’s latency outperforms DCSMSS + LEACH and SDS with the increased number of queries per batch.

#### 5.2.2. Range Query

**Figure 14:**
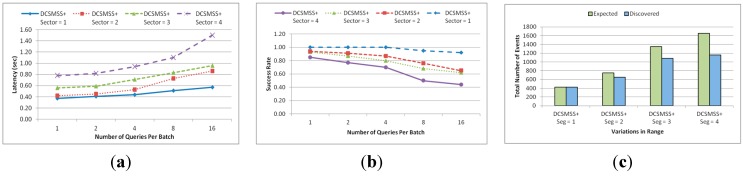
(**a**) Latency; (**b**) success rate and (**c**) total number of events.

This experiment was conducted in order to realize the performance of range query in various scenarios. The network size and number of queries was the same as the previous experiment. In Figure 14a–c, experiments were conducted for four different variations of range query. The range of the queries was varied in such a way so that in case one to four the number of sectors for the target data varies from one to four. For example, DCSMSS + Sector = 2 refers to the case where the target result of the query is to be fetched from two neighbor sectors. 

Figure 14a shows the average latency of each scenario as a function of number of queries per batch. As expected, the latency increases when the number of target sectors increases. If the target range of a query includes more than one sector all the corresponding SH fetch data from their respective segments and returns the data to the source SH. It is observed that, the latency for all scenarios grows slightly when the number of queries per batch increases except for the scenario DCSMSS + Sector = 4. In the case of DCSMSS + Sector = 4, latency begins to grow sharply when the number of queries increases from four to eight. This happens because of the congestion created due to the high number of reply packets flowing to the source query node from four neighbor sectors in response to a single query.

Figure 14b shows the success rate of each scenario as a function of the number of queries per batch. As expected, the performance of different scenarios is inversely proportional to the number of target sector. It is noted that all approaches falls slightly when the number of queries per batch increases from one to four but they start dropping sharply with the increase of number of queries per batch from four to sixteen. Figure 14c shows the number of discovered data items for each scenario when the number of queries per batch is four.

#### 5.2.3. KNN Query

**Figure 15:**
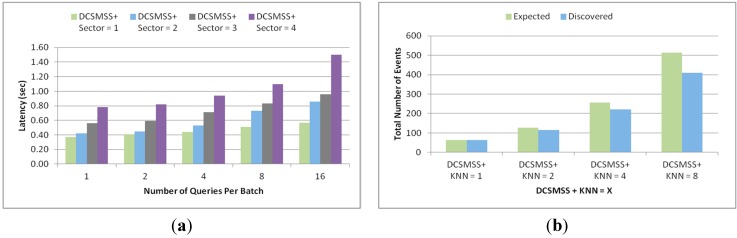
(**a**) Latency and (**b**) success rate.

The setting of this experiment was similar to that of the previous experiment. Like the previous experiment, the value of *k* in *KNN* (*q*, *k*) in the four different scenarios was varied in such a way that the target number of sectors varied from one to four. It is observed from Figure 15a that the latency is directly relative to the number of target sectors from which the resultant query is to be fetched. In addition, latency increases for each scenario with the increase in the number of queries per batch.

Figure 15b shows the total number of events finally discovered in comparison to the total number of expected events when the number of queries per batch is four. The discovery rate was 100% when the number of target sectors is one but it gradually falls with the increase in the number of target sectors. This happens due to the packet loss during the response time. When the number of target sector increases with regard to the increase of the value of *k*, the volume of reported events for single query increases significantly. This large number of reported events created hotspot and congestion around the query node and the corresponding relay SH of its route.

#### 5.2.4. Similarity Searching

**Figure 16:**
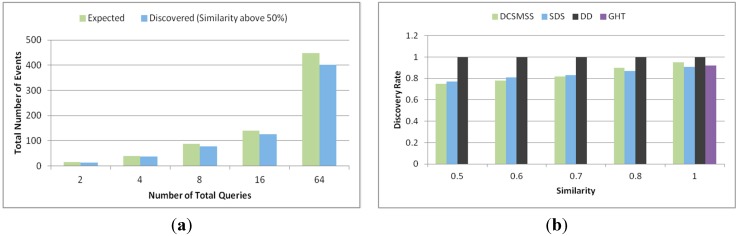
(**a**) Total number of events and (**b**) discovery rate.

The setting of this experiment was same as the previous experiment except the number of queries generated. The number of actual data items in the system and the number of discovered data items with no less than 50% similarity is shown in Figure 16a. This similarity is measured in terms of range query. After calculating *h_q_* of a query, *r* is calculated as ±0.25 *h_q_*. Thus, the range of the query was defined by [*h_q_* − 0.25 *h_q_*, *h_q_* + 0.25 *h_q_*]. The target head nodes where query was forwarded were *SH_j_*, *SH_j_*_+1_, …, *SH_k_*, where *P_j_* ≤ *h_q-r_* ≤ *P_j_*_+1_, *P_k_* ≤ *h_q+r_* ≤ *P_k_*_+1_ and *j* ≤ *k*. From Figure 16a it is observed that DCSMSS can always discover more than 85% of this type of data events.

Figure 16b shows the discovery rate of DCSMSS, SDS, DD and GHT in terms of similarity between the discovered data and the query. Discovery rate is defined as the percent of events that have certain similarity to a query and that can be discovered. In the second experiment, in total 100 queries were generated with four queries per batch. Since GHT is not locality preserving in data storage, its exact-mapping querying cannot locate similar data and thus for the GHT only 100% similar data is considered. Unlike other approaches, DD broadcasts queries to all SH and accordingly achieves 100% discovery rate. However, SDS and DCSMSS discover 85%~90% similar data. However, DCSMSS provide an optimized trade-off between energy consumption, latency and discovery rate.

#### 5.2.5. Scalability

**Figure 17:**
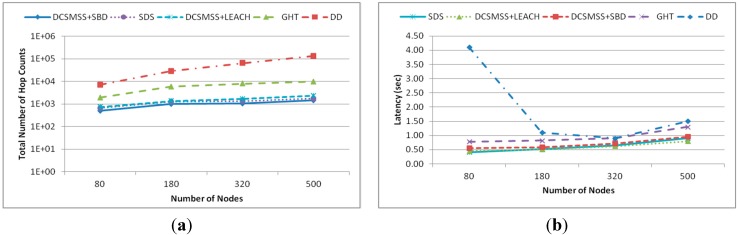
(**a**) Total number of events and (**b**) discovery rate.

This experiment was conducted in four different network field size of 60 × 60 m^2^, 90 × 90 m^2^, 120 × 120 m^2^ and 150 × 150 m^2^ containing 80, 180, 320 and 500 nodes, respectively. In total 200 queries were generated with eight queries per batch. Figure 17a shows the total number of hops. It demonstrates that DD’s total number of hop count is much higher than other approaches and grows sharply. This refers to the poor scalability of DD. The total number of hop counts for DCSMSS + SBD, DCSMSS + LEACH, SDS and GHT grows relatively slowly, which demonstrates the high scalability of these approaches. However, DCSMSS + SBD provides reasonably stable performance in terms of the total number of hops. This implies that this scheme has relatively stable routing performance for different size WSNs.

Figure 17b demonstrates the latency performance of each approach for different network sizes. DD has higher latency than other approaches with a dramatically higher latency when the network size is small (80 nodes). This happened because DD broadcasted the same number of queries in a small network creating high traffic with subsequent congestion in the network. In contrast, DCSMSS + SBD, DCSMSS + LEACH and SDS exhibit low latency across varying network sizes. This indicates the high scalability of these approaches.

**Figure 18:**
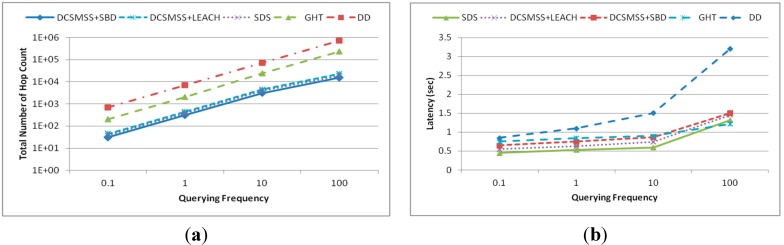
(**a**) Total number of events and (**b**) discovery rate.

Figure 18 illustrates the experiments which were conducted in a scenario of 120 m × 120 m rectangular field, in which 320 nodes are randomly and independently placed. These experiments were executed for 50 s with the querying frequency varied from 0.1 to 100 queries/s. Figure 18a,b show the total hop count and latency as the function of the querying frequency. Figure 18a demonstrates that the total number of hops for all approaches increases linearly. However, the performance of DD is lower because its broadcasting technique leads to vast traffic. It is also noted that the total number of hops for DCSMSS + SBD, DCSMSS + LEACH and SDS schemes is less than that of GHT. GHT always sends a query to ten different nodes for every attribute. SDS always sends queries to five sectors and DCSMSS sends to *i* sectors depending on the range *r*. That’s why DCSMSS based schemes show lower hops while SDS is slightly higher.

As shown in Figure 18b, the latency of each approach increases with an increase in the querying frequency. The latency of all approaches grows slightly with the increase in querying frequency from 0.1 to ten and then grows sharply when the frequency increases to 100 queries/s. It is also noted that DD has the highest latency. Since DD broadcasts queries to all sectors it generates congestion, packet loss excessive retransmission. Despite having lower collision and subsequent packet loss and retransmission, DCSMSS + SBD’s latency is higher than SDS and DCSMSS + LEACH due to the reasoning explained in Section 5.1.2. However, it is interesting to note that the latency of GHT is lower than other approaches when the querying frequency is 100 queries/s. This happens because the number of sectors is lower than the number of nodes and under heavy traffic routing relying on SH became more congested than routing relying on nodes. Moreover, the routing, referred to GPSR, used in GHT uses the greedy forwarding technique which eventually selects the shortest path to route packets.

## 6. Conclusions and Future Work

In this paper a highly scalable distributed information service, DCSMSS, is presented that provides improved performance over comparative schemes. The scheme is an efficient similarity search mechanism for WSN. DCSMSS was applied to a range of WSN scenarios utilizing modeling, simulation and a statistical analysis and found to provide lower latency and improved search accuracy when compared to relatively recent alternate approaches. Discussion has been provided surrounding the alternate approaches and the improvements found when DCSMSS is applied. The research is continuing with future work considering methods to reduce complexity and improve processing at the nodes and SH to reduce energy utilization. DCSMSS has been simulated with a static, non-mobile network. Problems are expected when applying virtual sector formation or synchronization to groups of mobile nodes. Virtual sector or cluster formation in the dynamic WSN is an interesting area for future research. Furthermore, in current model, SH is the only gateway to the sector and hence it could create hotspot around the SH. This issue can be resolved in future work by outsourcing some of the responsibility to MNs, which will act as Secondary SH (SSH). A prototype implementation of DCSMSS is under development using the Texas Instruments’ (TI) CC2530 Evolution Module (CC2530EM) [17], which is ZigBee/IEEE 802.15.4 compliant System-on-Chip with an optimized 8051 MCU core and radio for the 2.4 GHz unlicensed ISM/SRD band. 
